# Mobility and growth in confined spaces are important mechanisms for the establishment of Bacillus subtilis in the rhizosphere

**DOI:** 10.1099/mic.0.001477

**Published:** 2024-08-06

**Authors:** Ilonka C. Engelhardt, Nicola Holden, Tim J. Daniell, Lionel X. Dupuy

**Affiliations:** 1 Department of Geosciences, University of Tuebingen, Tuebingen 72074, Germany; 2 Department of Rural Land Use, Scotland’s Rural College, Aberdeen AB21 9YA, UK; 3 Molecular Microbiology: Biochemistry to Disease, School of Biosciences, The University of Sheffield, Sheffield S10 2TN, UK; 4 Department of Conservation of Natural Resources, Neiker, Derio 48160, Spain; 5 Ikerbasque, Basque Foundation for Science, Bilbao 48009, Spain

**Keywords:** *B. subtilis*, mobility, plant–microbe interaction, root colonization, soil microhabitats

## Abstract

The rhizosphere hosts complex and abundant microbiomes whose structure and composition are now well described by metagenomic studies. However, the dynamic mechanisms that enable micro-organisms to establish along a growing plant root are poorly characterized. Here, we studied how a motile bacterium utilizes the microhabitats created by soil pore space to establish in the proximity of plant roots. We have established a model system consisting of *Bacillus subtilis* and lettuce seedlings co-inoculated in transparent soil microcosms. We carried out live imaging experiments and developed image analysis pipelines to quantify the abundance of the bacterium as a function of time and position in the pore space. Results showed that the establishment of the bacterium in the rhizosphere follows a precise sequence of events where small islands of mobile bacteria were first seen forming near the root tip within the first 12–24 h of inoculation. Biofilm was then seen forming on the root epidermis at distances of about 700–1000 µm from the tip. Bacteria accumulated predominantly in confined pore spaces within 200 µm from the root or the surface of a particle. Using probabilistic models, we could map the complete sequence of events and propose a conceptual model of bacterial establishment in the pore space. This study therefore advances our understanding of the respective role of growth and mobility in the efficient colonization of bacteria in the rhizosphere.

## Introduction

The rhizosphere, the area of soil which is influenced by the plant root, is a hotspot of bacterial abundance and activity in the otherwise rather barren bulk soil. The plant may exude as much as 20–40 % of its photosynthate which is a vital supply of labile C to fuel soil microbial processes [1] and is critical in shaping the composition of the root microbiome [2]. The root microbiome in turn can have profound effects on the plant health and productivity through its effects on nutrient acquisition, priming of plant defences and protection against pathogens [2,4]. This has led to the shift towards potential use of bio-fertilization and bio-control practices in agriculture, effectively manipulating the root microbiome towards more plant growth–promoting species to take over the function of chemical fertilization and pesticide applications. Unfortunately, gaps still exist in our understanding of the plant–microbe interactions in soil, particularly how microbes are able to communicate and compete along the many varied microsites along the root, which vary not only in space [5] but also in time [6,7].

Previous studies have shown that spatially defined root exudation patterns result in distinct microbial communities associated with different root zones [8]. Some studies highlight the root elongation zone as a major source of exudation and thus hotspot for microbial interactions [9] and an area of high colonization for *Bacillus subtilis* [10], a common model of Gram-positive plant growth–promoting rhizobacterium. Similarly, the root cap, with its regular sloughing of cells and high polysaccharide-containing mucilage secretion, has been highlighted as a zone of interest for soil microbes [11]. However, since the root cap is in constant motion through the soil, the question remains as to whether bacteria can move with the root tip. Additionally, any microsites on the root where there is a localized increase in nutrient ‘leakage’ from the root due to weakness or discontinuity of the epidermis, increased microbial activity and colonization may be observed. This includes sites of transient damage during lateral root emergence or physical damage to the epidermis [12] and root cell junctions [13].

In turn, microbes in the soil need to be metabolically active, be able to detect the plant exudate signals and move towards them, compete for root niches if necessary and successfully attach to the root surface for successful rhizosphere colonization [8]. Even though passive movement of microbes in soil exists, studies have shown that active movement coupled with chemotaxis is vital for successful colonization [2,14]. Once they have successfully navigated the complex soil matrix and have arrived at the root, rhizosphere microbes need to interact with and attach to the root surface [2,15]. Additionally, they need to outcompete other microbes and possibly deal with plant defence mechanisms [16].

The successful colonization of the root by rhizosphere bacteria therefore depends on a number of mechanisms and interactions which are likely susceptible to the pore structure surrounding plant roots. In this study, we aim to break down how some of the steps involved in early root colonization are influenced by the pore structure. We developed a model system composed of live lettuce seedlings, growing in transparent soil with fluorescently labelled *B. subtilis*, coupled with high resolution, time-lapse microscopy and image analysis techniques. We hypothesize that bacteria are not only attracted to specific rootzones but that in a soil system, the interaction between soil particles and the root may lead to heterogenous microhabitats with different environments across very small distances.

## Methods

### Transparent soil and imaging microcosms

**Fig. 1.**
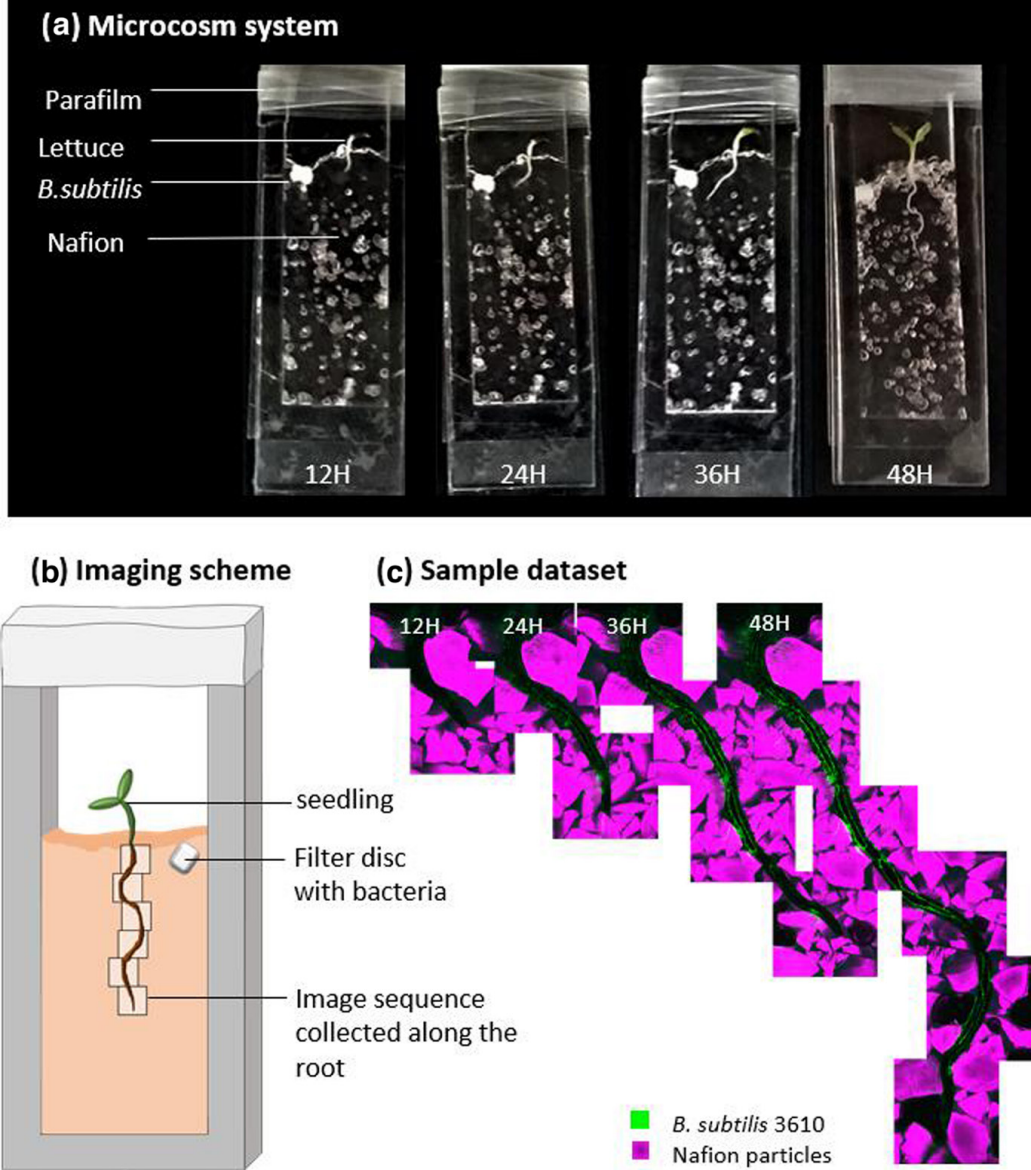
Live imaging of the dynamic patterns of microbial activity in soil during early colonization of the root. (**a**) Custom-made microcosms containing lettuce seedlings and fluorescently labelled *B. subtilis* in transparent soil (Nafion) were grown for up to 48 h. (**b**) Individual Z-stack images were collected along the root at each timepoint. (**c**) Max-projection of Z-stacks was aligned to show the root (unstained), *B. subtilis* (green) and soil particles (Nafion, pink) over time.

Transparent soil was prepared from Nafion precursor beads (Ion Power Inc., USA) as previously described by Downie *et al*. [17] with minor modifications (see Liu *et al*. [18] for details on Nafion preparation). In short, Nafion pellets were broken down to particle sizes of 250–1250 µm using in a cryogenic mill (SPEX SamplePrep 6770). Following this, the particles were converted to anionic form by soaking in 6 M KOH, 35 % 5 M DMSO at 80 °C, followed by 3 M nitric acid at room temperature before titrating with Hoagland’s basal medium (Sigma M5519) at 4.4 g L^−1^. Sulphorhodamine B was added to the Nafion particles to enable imaging the soil particles with a 561 nm laser. Transparent soil was sterilized by autoclaving for 20 min at 121 °C before use. The custom-made microcosms (Fig. 1a) comprised of a three-sided polydimethylsiloxane frame (SYLGARDTM 184 Silicone Elastomer Base; length 50×outside width 25 and inside width 20×thickness 3 mm) between two standard microscopy glass slides (VWR, microscope slides at length 76×width 26×thickness 1 mm) (see Liu *et al*. [18] for details on chamber construction).

### Plants and microbes

Lettuce (*Lactuca sativa*) seeds were surface sterilized (10 % bleach for 15 min) before germination on distilled water agar for approximately 3 days (until 2 mm root visible). A single seedling was then transferred to each sterile microcosm which was pre-filled with transparent soil and saturated with percoll.

Fluorescently labelled (GFP) wild-type *B. subtilis* strain NRS1473 (Table S1, available in the online Supplementary Material [19]) was grown in MSgg medium (5 mM KH_2_PO_4_ and 100 mM MOPs adjusted to pH 7.0, supplemented with 2 mM MgCl_2_, 700 µM CaCl_2_, 50 µM MnCl_2_, 50 µM FeCl_3_, 1 µM ZnCl_2_, 2 µM thiamine, 0.5 % glycerol and 0.5 % glutamate) for about 24 h at 18 °C, while shaking at 200 r.p.m. (increase in OD_600_ of 1–1.5 orders of magnitude). After incubation, the MSgg solution was replaced with Murashige and Skoog medium to remove any carbon source before inoculating approximately 2 million c.f.u. onto a sterile filter disc which was inserted into the microcosm level with the root (Fig. 1a). All microcosms were sealed on the top using parafilm, to prevent excessive drying and contamination. This is the time 0 (T0) for the biological system. The microcosms were incubated in a near vertical position (at approximately 30° angle) in a growth cabinet at 21 °C (light cycles of 16 h light to 8 h dark) and were only removed and temporarily placed horizontally for image acquisition at 12, 24, 36 and 48 h post-inoculation (T0).

### Timeline image acquisition

Images were collected using a confocal laser scanning microscope (Nikon A1R) with a 488 nm Argon laser (40 mW) and a 561 nm Sapphire laser (20 mW). A Nikon 4× plan fluor objective was used for the time-series image collection, with 6.21 µm per pixel resolution. Individual image stacks (Z-series) of 3.2×3.2 mm with an average of 227.6±66 µm in depth were collected along each root (Fig. 1b, c). Depending on root length, two to six image stacks were collected, from the tip to the mature root hair zone. Roots, which failed to grow, showed no bacterial colonization or were too obscured by soil particles for imaging, were excluded. This resulted in a total of 12 roots from three separate runs which met these criteria over the whole time period and could thus be tracked. The image data used in this study are available on FigShare repository (https://doi.org/10.6084/m9.figshare.25225517).

### Image analysis

The image dataset was processed to quantify bacterial abundance both on the root surface and in the soil. Images were first segmented using the Weka segmentation in ImageJ [20]. We applied the segmentation with five classes, namely, soil particle, pore space, mobile bacteria (MB), bacterial biofilm on root (RB) and bacterial biofilm on soil particles (PB). No root staining was performed, so the region occupied by the root was drawn manually on the image. In the next step, binary image data obtained by the segmentation of the image were broken down into small regions using a watershed algorithm. Each region identified was used to extract the mean fluorescence intensity from the image and was used to determine the probability distribution functions of bacteria in the pore space (see the following section). The distance from the root surface or from the surface of the particle was computed using the distance transform of the binary image. Pixel intensity was calibrated against bacterial cell numbers using images where cell density was low and it was possible to count individual cells from the image. A relationship between cell number and total pixel intensity was determined (Fig S1) as pixel intensity=39*cell number+331 which was used to compute cell density along the root.

### Probabilistic model

The image analysis programmes provide a collection of subregions of the soil domains together with a description of the nature of the population in this subregion, e.g. root biofilm (RB), particle biofilm (PB) and mobile bacteria (MB). Such data do not easily translate into a synthetic representation of how the pore space is utilized and when. To address this issue, we developed a probabilistic modelling approach to build maps of the likelihood of a bacterial cell to occupy certain regions of the pore space through time. The position of the bacterium in the pore space is described as a function of the distance from the surface of a particle (noted $d_{p}$), the distance from the root surface ($d_{r}$) and the distance from the root tip ($d_{a}$), and together, they are denoted as a positional vector x. Since there is no *a priori* knowledge on how the bacterium should distribute in such the space of vector x, we employed a non-parametric modelling approach to construct density distribution functions.

More formally, we consider that the probability *P* that a bacterium introduced in the system is found at position x in the pore space. Because the position in the pore space is defined as a continuous variable, the probability *P* is defined as a function of the probability density distribution function *p*, such that

A more general definition must be given because several parameters must be used to describe the covariates x defined in a multidimensional space Ω:

Here, $x=\left[x_{1},\ldots ,x_{n}\right]$ is a multidimensional vector that denotes position in the pore space such as the distance from the surface of the particle (noted $d_{p}$), the distance from the root surface ($d_{r}$) and the distance from the root tip ($d_{a}$). An estimation of the probability density distribution function $\tilde{p}(x)$ is obtained from *N* patches of bacteria detected by image analysis and characterized from their area $\left(A^{i}\right),i\leq N$ and their average pixel intensity $(I^{i}),i\leq N$ . The kernel density estimation of the probability density function $\tilde{p}$ is defined as

*K* is the kernel function, a function decreasing with the distance from the position $x^{i}$ where a patch of bacteria is detected with ${∭}_{R^{n}}K\left(x\right)dx=1$ and *M* is the total quantity of bacterial fluorescence recorded in the dataset:

so that ${∭}_{R^{n}}\tilde{p}\left(x\right)=1$. $h^{i}$ is a parameter vector that defines the width of the kernel, and its value is set to the radius of the *i*th patch of bacteria detected by image analysis ($h^{i}=\sqrt{A^{i}/\pi }$).

Since patches of bacteria have a finite support and only the mean density was recorded, Parzen windows (or top hat functions) were used. Parzen windows defined an ellipsoid in the *n*-dimensional space where the value returned by the function is constant:

where *C* is the normalization constant that insures K has an integral of 1, i.e. $\frac{\pi^{k}}{k!}\prod_{j=1}^{j=2k}x_{j}/h_{j}$ for n=2k and $\frac{2k!{\left(4\pi \right)}^{k}}{(2k+1)!}\prod_{j=1}^{j=2k+1}x_{j}/h_{j}$ for n=2k+1. It can easily be verified that the definition of $\tilde{p}$ satisfies the condition for probability density function in equation 2 and that the relationship captures the increase in probability of the microbes in regions where patches are more frequent and/or of higher fluorescence intensity.

We also determine the mean bacterial cell density $\rho \left(x\right)$ as the product of the probability density function by the average quantity of bacteria measured in the rhizosphere:

R is the number of roots in the dataset. To establish the spatio-temporal maps of bacterial distribution of the root colonization, we therefore extend the estimation of the bacterial density distribution to

Using this method, we established the regions in the pore space most likely to be colonized by the bacteria. We also used the same method to determine the null model. In this case, the segmentation of the pore space is used to determine the probability density distribution in the case where all regions of the pore space have equal probability to be colonized.

All the scripts used to process image data and compute probability density distribution functions are available on the GitHub repository (https://github.com/LionelDupuy/WEKASegBacteria/). The programmes consist of an ImageJ [21] plugin for automating the application of Weka segmentation [20] to the dataset, various ImageJ scripts for extracting features in the image and a Python script for reconstructing bacterial cell density. All figure data used to obtain the results are available at the FigShare repository (https://doi.org/10.6084/m9.figshare.25225517).

## Results

### There are different patterns of bacterial activity in soil

**Fig. 2.**
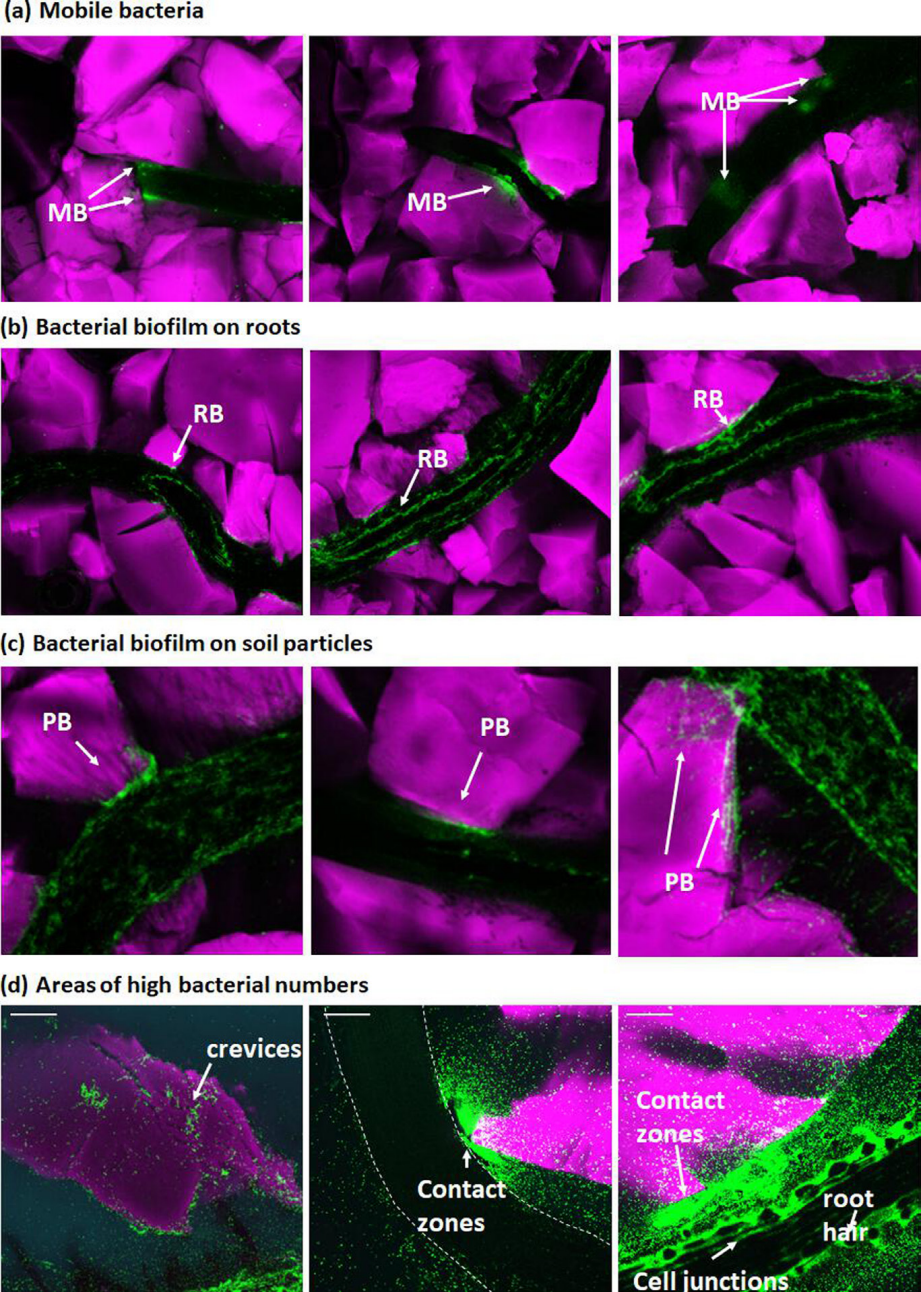
Live imaging reveals the following: (**a**) MB are characterized by fuzzy areas of fluorescence. They are observed early during the colonization process, particularly in confined spaces between the soil particles and the root surface. (**b**) RB is observed later during the colonization process. (**c**) SB is also observed later during the colonization process. (**d**) High-resolution images (20× objective, scale bar=100 µm) show that areas of high bacterial activity (mobile and biofilm) are associated with soil particle crevices and root particle contact zones, in root cell junctions and root hair emergence sites.

MB and adherent biofilms had different visual characteristics, detectable in composite images (with a 4× objective) (Fig. 2). While MB resembled hazy clouds (Fig. 2a), bacterial biofilm showed more defined borders and patterns that likely correlated with surface topography of the root (Fig. 2b) and biofilms on soil particles appeared associated with the topography of the surface of the particle (Fig. 2c). Live observation of microbial clouds (Fig S2) confirmed that the clouds were made up of mobile cells with different swimming directions. Different niches were detected by higher-resolution microscopy (20× objective) showing a propensity for crevices in soil particle (Fig. 2d, left) and root–soil particle contact zones (Fig. 2d, middle) as well as regions of biofilm formation in root cell junctions and around root hairs (Fig. 2d, right). The complete set of data used in this study can be examined in https://doi.org/10.6084/m9.figshare.25225517.

### Image analysis allows quantification of the distribution of bacterial activity in the rhizosphere

**Fig. 3.**
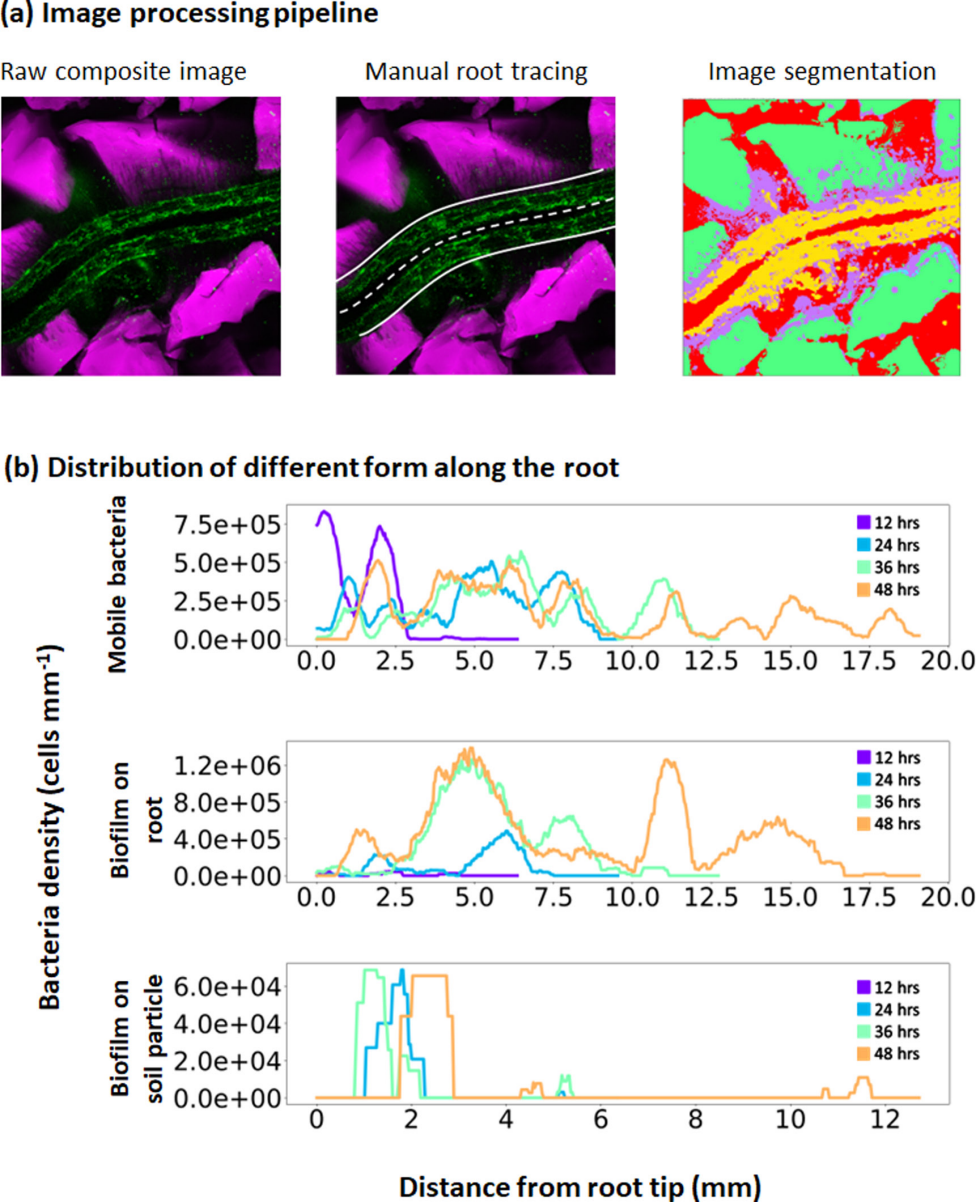
Quantitative analysis of image data along the root over time. (**a**) Image processing steps consist of assembling a coloured image data regrouping the fluorescent channels for roots and particles (left) and manually tracing the centreline (white discontinuous line) and boundaries (white, solid line) of the root (centre). Image segmentation is performed using Weka trainable algorithm (right) using five classes (MB, biofilm on root, biofilm on soil particle, soil particle and pore space). Purple, yellow, blue, green and red colours indicate pixels of the image occupied by mobile bacteria, biofilms on root, biofilm on soil paticle, soil particle and pore space respectively. (**b**) Post-processing of the data then allows extracting the distribution of bacteria. Here, typical profiles for MB (top), bacteria that form biofilms on the root (middle) or bacteria that are attached to soil particles (bottom) as a function of the distance from the tip.

Image data from confocal microscopy (Fig. 3a, left) was used to extract key features of pore space, root morphology, microbial patterns and association with root surfaces or soil particles. First, the root was manually segmented using polygonal shapes (Fig. 3a, middle). Then, using a trainable image segmentation tool, it was possible to differentiate not only between MB and biofilm bacteria but also between biofilm on root and biofilm on soil particle. The image data generated consisted of an image stack with each slice containing the probability that the pixel belongs to each class. Using the maximum probability for the four segmented classes, it was possible to determine whether a pixel in the image belonged to a particle, a mobile population of bacteria, a biofilm on the root or particle and the pore space (represented with different colours in Fig. 3a, right). By relating the total number of pixels identified by the segmentation to the detection position, it was possible to establish dynamic maps of bacterial presence in the rhizosphere. Observation of the raw data generated by such analyses revealed that the presence of bacteria on the root was both sparse and highly dynamic (Fig. 3b).

### Colonization of the root follows a sequence of events

**Fig. 4.**
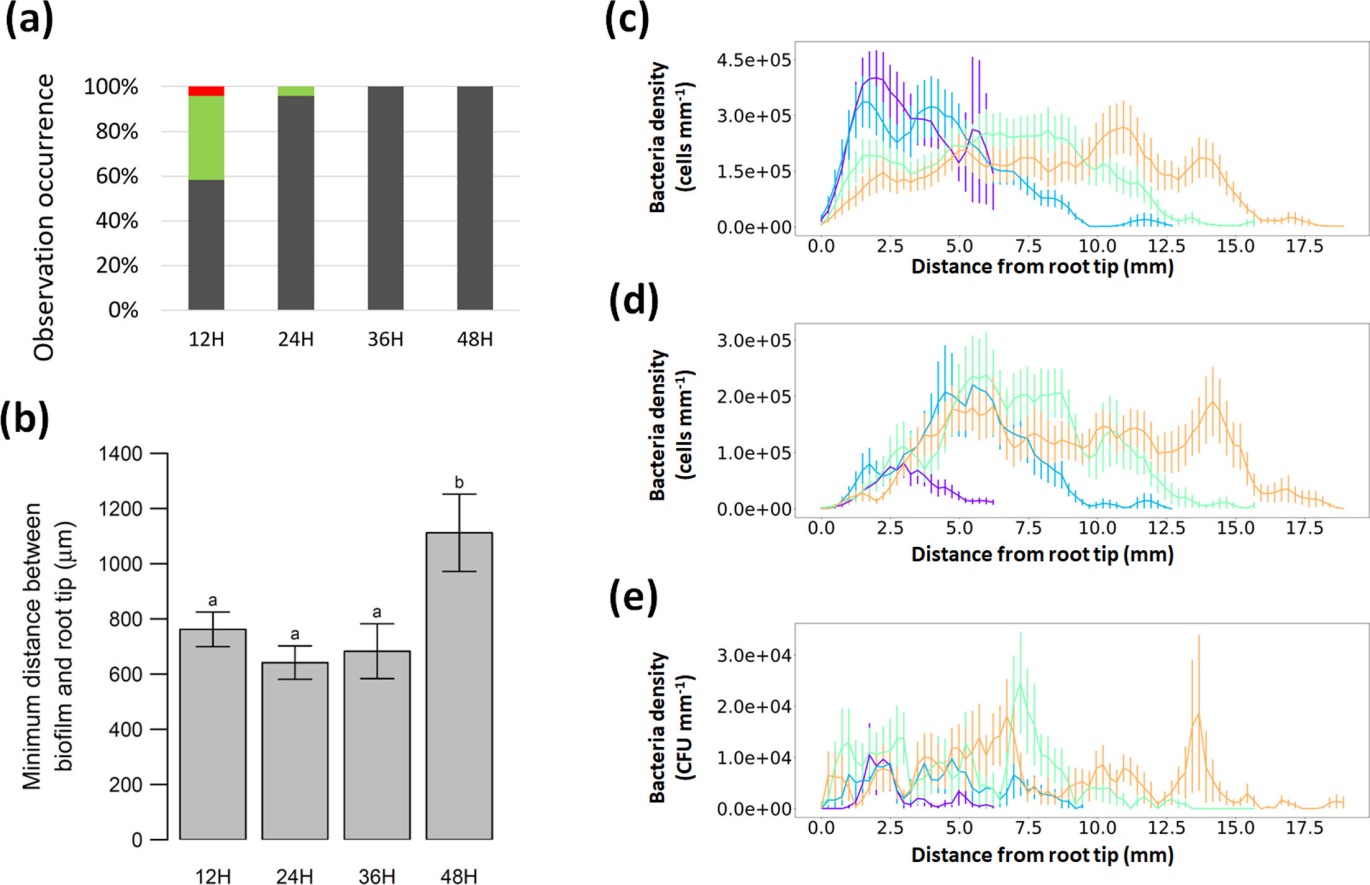
Bacterial distribution in space and time. (**a**) Qualitative analysis of the image dataset showed that the colonization of the root follows a clear sequence over time. Biofilm could be detected on roots as early as 12 h post-inoculation. Images with MB showing no evidence of biofilm formation were also observed in earlier time points. Red indicates the percentage of images where no bacteria observed, green the percentage where only mobile bacteria are observed, grey he percentage where both mobile and biofilm bacteria are observed. (**b**) Biofilms formed on the root but appeared to be excluded from the extreme root tip. Biofilm was found at least 794.26±384.89 µm from the root tip at any of the observed time points. This was particularly prominent at 48 h where the minimum distance between root tip and biofilm was significantly higher than at any other time (*P*=0.005). (**c–e**) A more detailed analysis of the image dataset showed that the colonization of the root follows a clear sequence. The bacterial density is shown in colony forming unit per unit length of root (CFU mm^-1^). (**c**) First MB appear at the tip of the root during the first 24 h post-inoculation. Following this peak of activity, MB were detected uniformly along the root in lower quantity, with lower presence near the root tip. (**d**) RB was observed further away from the root tip even at 12 h post-inoculation. The colonization of the root appeared to be uniformly distributed on mature tissues. (**e**) The colonization of the particle surface appeared to increase with time, but no clear patterns could be observed.

First, we visually classified the nature of the colonization (Fig. 4a) depending on the degree of root colonization as a function of time. The relative proportion of the types of populations was found to change through time. MB together with the absence of colonization of the root was mostly observed around the 12 h time point, and it was often accompanied or followed by the colonization of the root (Fig S3). Some samples however did not show significant colonization of either soil particles or roots within 12 h (Fig. 4a). We also looked at which part of the root was colonized by the bacteria and could observe that the extreme root tip was poorly colonized in all samples (Fig. 4b). Biofilms on the root were consistently observed at a distance of at least 794.26±384.89 µm from the root tip and were significantly further from the tip at 48 h than at the preceding time points.

To characterize the different steps needed for the bacterium to establish in the rhizosphere or on the root, we reconstructed the bacterial cell density distribution function of the three types of bacterial populations mobile bacteria (Fig. 4c), root biofilm (Fig. 4d) or particle biofilm (Fig. 4e)] along the root over time. The analysis confirmed that MB are initially detected around the root tip 12–24 h after inoculation. MB reached a peak of approximately 1.5×10^7^ cells per millimetre of root on average (Fig. 4c). The density of MB subsequently reduced while the bacterial cell density in the form of biofilm on the root increased over time. While the overall number of cells in the form of biofilm increased through time, locally, the cell density reached a plateau at about 8×10^6^, 24 h after inoculation, and subsequently expanded on new root tissues (Fig. 4d). In contrast, bacterial biofilms on the soil particles tended to increase with time did not show any clear spatial pattern (Fig. 4e).

### Soil pore structure creates niches for bacteria

**Fig. 5.**
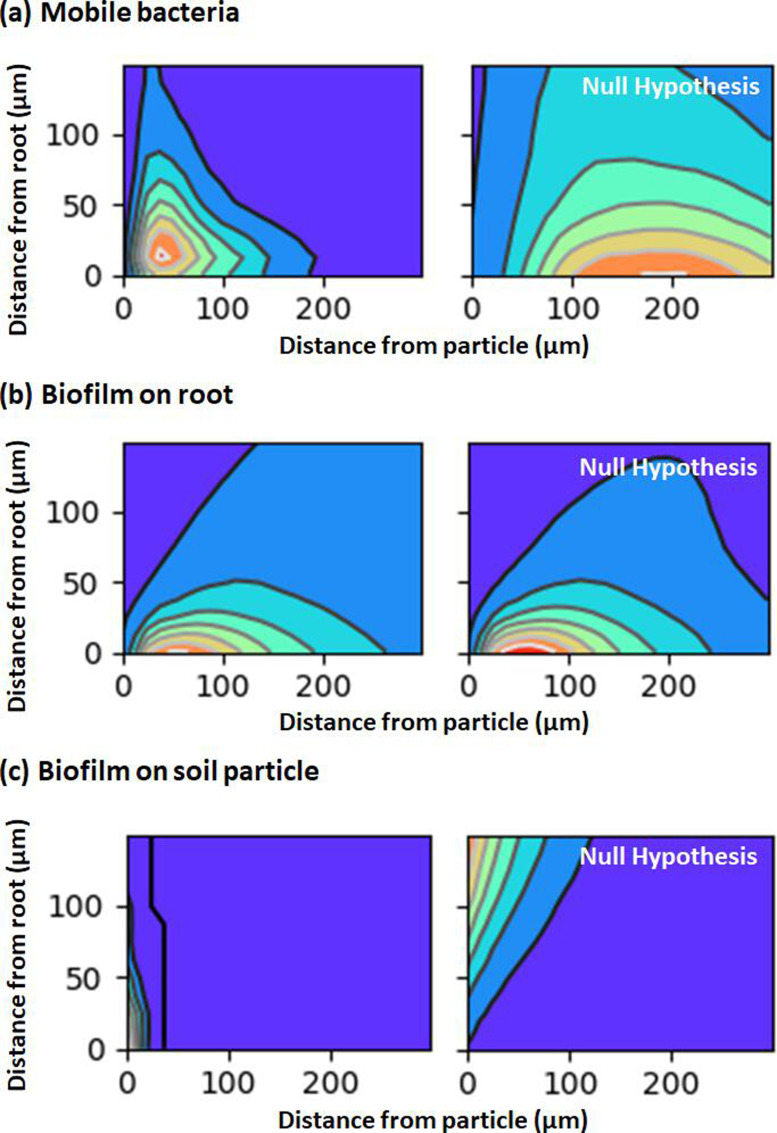
Probability density distribution maps show the regions of the pore space most likely to be occupied by *B. subtilis*. (**a**) MB are primarily observed in confined spaces both close to the surface of the root and that of a soil particle (left). MB were also observed up to 200 µm from the particle when close to the root surface or up to 150 µm when close to the surface of a soil particle. The observed pattern was not random as shown by a similar plot for pixels of the pore space (null hypothesis, right). (**b**) RB were observed at a range of distances from the particles, and the pattern did not differ from the distribution of root pixels (null hypothesis, right), suggesting that the bacteria occupied the root surface indifferently. (**c**) Biofilms on soil particles could be observed on the surface of the particles up to 100 µm distance from the root surface (left). By comparison, the distribution of pixels on the soil particles exhibited at greater distance both from the root and from the surface of the particles (null hypothesis, right).

In order to assess whether the pore structure creates microenvironments that are more favourable to bacterial growth than others, the image segmentation algorithms were used to determine the likelihood of colonization of the bacteria considering their location in the pore space and proximity to the root (Fig. 5). We coupled the manual root tracing of the root surface and centreline to Euclidean distance maps to construct probability density distributions of bacteria in the soil domain as a function of time. The probability density distribution maps revealed that MB were distributed predominantly in areas which were either within 200 µm from the root or from a soil particle (Fig. 5a, left). The combination of proximity of both root and particle surface appeared to create optimal environments for the moving bacterium to grow, with the vast majority of bacteria measured in these environments. The distribution was not random, since by comparison, the distribution of pixels from the pore space (Fig. 5a, right) was distributed more uniformly across a broader range of distance from root and particle surfaces. On the root surface, no distinguishable pattern could be detected between biofilm bacteria and pixels (null hypothesis), revealing no significant relationship (Fig. 5b). Biofilm on soil particles could be found as far as 100 µm from the root, again, in a non-random distribution (Fig. 5c). The distribution of pixels on particles (null hypothesis) was observed at greater distance not only from the root surface but also from the particle surface.

## Discussion

### Soil–root interactions may create hotspots of *B. subtilis* colonization

Microbial biodiversity in the rhizosphere is believed to result from the formation of multiple microhabitats segregating the pore space into contrasting environments (in terms pH, oxygen concentration and water content in particular), and these in turn promote a greater diversity of microbial functions [22,23]. Unfortunately, experimental validation of such hypotheses has proven difficult since making such observations in natural soils is not possible [24].

High-resolution, live imaging techniques coupled with mathematical modelling have enabled us to visualize and describe, at the micro-scale, the spatial and temporal distribution of *B. subtilis* during the early colonization of roots in a soil-like matrix. Our results confirm a large variation in microhabitats and niches within our relatively simple 2 ml microcosm system. We identified a highly heterogenous ecosystem, with hotspots for colonization with a distinct propensity for the confined spaces created by root surface and soil particle contact zones. We hypothesize that these confined spaces offer a suitable environment for bacteria due to a physical sheltering effect of confined spaces [25] as well as the likely high abundance of labile nutrients exuded from the root into this zone. This may be further enhanced due to micro damage of the root surface caused by rubbing against the particle surface and possible cellular release [12]. Such interaction between solid soil particles and the root surface creates a more heterogenous environment, leading to colonization patterns that are even more irregular or patchy than those observed in microfluidic studies [10].

While the mechanisms of bacterial cell interactions with the root were observed in an artificial soil–like medium, numerous questions remain as to how these mechanisms vary when operating in a natural soil environment. A number of factors are likely to be affected when changing the nature of the granular substrate in which plant and microbes are growing. This includes, but is not limited to, the ability of the exudates to bind to soil particles, the quantity and quality of binding sites, the rate at which root exudates are degraded by the biota, changes in soil porosity and variation in connectivity of the pore space [26,29]. Therefore, model systems such as those used in this study are complementary to experiments in natural soils.

### Root–bacteria interactions are specific to the location in the rhizosphere

Bacterial species have different abilities to colonize the rhizoplane [30,31]. In our experiments, we observed that attachment and proliferation of bacteria were excluded from the surface of the extreme root tip, which is consistent with the findings reported in the literature where colonization of *B. subtilis* on the roots of different plant types was reported on elongated cells [32,38]. In the surrounding soil, however, bacterial cells were observed in the proximity of the root tips while attached and forming biofilms on the surface of the particles. The analysis of bacterial cell density also confirmed this observation (Fig. 4). It can be concluded that attachment and proliferation of the bacterium on the rhizoplane were influenced predominantly by cell type–specific interactions through cell-specific attachment mechanisms, e.g. adhesins [39,40], and/or due to responses from the plant immune system [41].

Root exudates are a source of labile nutrients for a multitude of bacterial species [42,43]. Since we could not detect specific patterns of colonization on the soil particles along the root, we hypothesize that the proliferation of bacteria on soil particles is related to the chemical composition of the soil resulting from the accumulation of rhizodeposits through time. In a natural soil, secreted root exudates can diffuse through the pore space to a limited distance from the root surface, usually within few millimetres from the root [7]. Additionally, the lifetime of the dissolved molecules in soil is limited when subjected to degradation by biological activity (half-life of few hours [44]). In our experiments, the bacterial abundance declined as a function of the distance from the root surface (Fig. 5a) with no colonization observed at distances exceeding 1 mm from the root surface, which is a short distance considering that the pore sizes were large in comparison with those of natural soils. It is likely that the diffusion of root exudates was limited by absorption at the surface of the soil particles which resulted in reduced diffusivity. Nafion, the material used for the fabrication of soil particles, is characterized by a strong ionic strength [45], and it has been shown to bind polysaccharides from root exudates [46].

### Chemoattraction sites and colonization sites do not coincide

The dynamics of mobile *B. subtilis* populations gave important information about the sequence of events that precede establishment on the surface of the root and on that of the soil particles. Twelve hours after inoculation, MB were primarily found surrounding the tip of the root. Since chemotaxis is orientated along the steepest chemotactic gradients, the convergence of MB towards the tip of the root may be indicative of where chemoattractants are in highest concentration. It is not surprising for such sites of high concentrations to occur close to the root tip since exudation rates are usually higher in this location [47]. In a recent study, it was found that manipulating the exudate composition at the root tip had an effect on the microbiome of the whole root system [48]. The site of emergence of lateral roots is other hotspots of root exudates, but these sites were not present in our experiment because of the short experimental duration (usually, several days are needed for lateral root initiation and emergence).

The accumulation of MB near the root tip, however, did not result in significant attachment and colonization of the root tip itself. It is possible that the biofilms recorded on the older portion of the root originate from the attachment and proliferation of cells at the root tip (root cap and cell division zone), since proliferation on root tissues has been observed in other model systems [49]. It is nevertheless unlikely to be a dominating factor in our experiment. First, the root cap does not facilitate the colonization of the epidermis since its cells detach and can subsequently disperse in the soil [50]. Experiments in hydroponic conditions, where bacterial suspensions are well mixed, showed a preference for attachment on the root elongation zone [10], and quantification of the relative contribution of attachment and proliferation of bacterial cells on the root surface has shown that colonization of the root surface is initially dominated by attachment rather than proliferation [42].

Since colonization of the root surface was likely dominated by attachment rather than proliferation, i.e. many bacterial cells present on the rhizoplane have not yet divided [42], a mismatch exists between the sites where the bacterium is attracted and the locations on the root that the bacterium can colonize. This mode of colonization may be suboptimal for the colonization of an immobile host, but it is unclear whether it confers a benefit when the host is moving. New root tissues originate from the quiescent centre which is located at the tip of the root. We can postulate, therefore, that the proliferation of bacterial cell density at the root tip may confer a competitive advantage, allowing the bacterial population to be present in greater abundance when the tissue is differentiating, and attachment becomes possible.

### Heterogeneity of the colonization is linked not only to soil microstructure but also to collective movements

Quantitatively, the analysis proposed in this study has determined the average cell densities (Figs.4  5) of the bacterium in various compartments of the rhizosphere and at different times following inoculation. However, the distribution of cell density was heterogeneous. This heterogeneity was due in part to the structure of the pore space. Bacterial cells preferentially occupied the smaller pore spaces (Fig. 5a) which is a known protection mechanism against predators and environmental stresses [25,51]. The close proximity of these locations to the root surface was also potentially due to the availability of nutrients.

The suitability of the habitat was not the only factor determining the heterogeneity of the bacterial cell density in the system. Large numbers of pores that were predicted to be optimal to MB remained unoccupied. More importantly, MB were observed in the form of clouds whose morphologies differed from the travelling fronts of bacteria usually observed in liquid and gel cultures [52] or predicted by mathematical models [53]. These results indicate that collective movements of bacteria may accentuate the heterogeneity of the bacterial cell density distribution in soil. For example, it has recently been shown that *B. subtilis* can form flocks when moving through soil [54]. Quorum sensing and attraction to kin, when occurring during movement through a heterogeneous substrate, may cause bacterial populations to leave physically accessible pores unexplored. This form of movement, in turn, adds to the heterogeneity of the distribution of attached bacteria. It was observed, notably, that groups of MB colocalized with the site of the formation of an early biofilm either on the root or on nearby soil particles (see Fig. 2a).

### Different steps of colonization of the rhizosphere

The high-resolution live imaging techniques have enabled us not only to describe the spatial patterns of *B. subtilis* around the young plant roots at the micro-scale but also to trace the movement and colonization over time. Live imaging in soil is still limited due to its nature with observational studies of bacterial colonization of roots grown in soil therefore still mostly unavailable. Though a lot of important information on microbial movement and biofilm formation has been gained from the large body of work performed in hydroponics and microfluidics (e.g. [10]), the complexity created by the physical structure of the soil network is inherently missing. Not only are the microhabitats in microfluidics device less heterogenous but also microbial movements are less constrained. Soil is a complex physical matrix where the variation in connectivity of the pore space and diffusion of chemoattractants may lead to the formation of locations on the root where the bacteria are not able to establish.

**Fig. 6.**
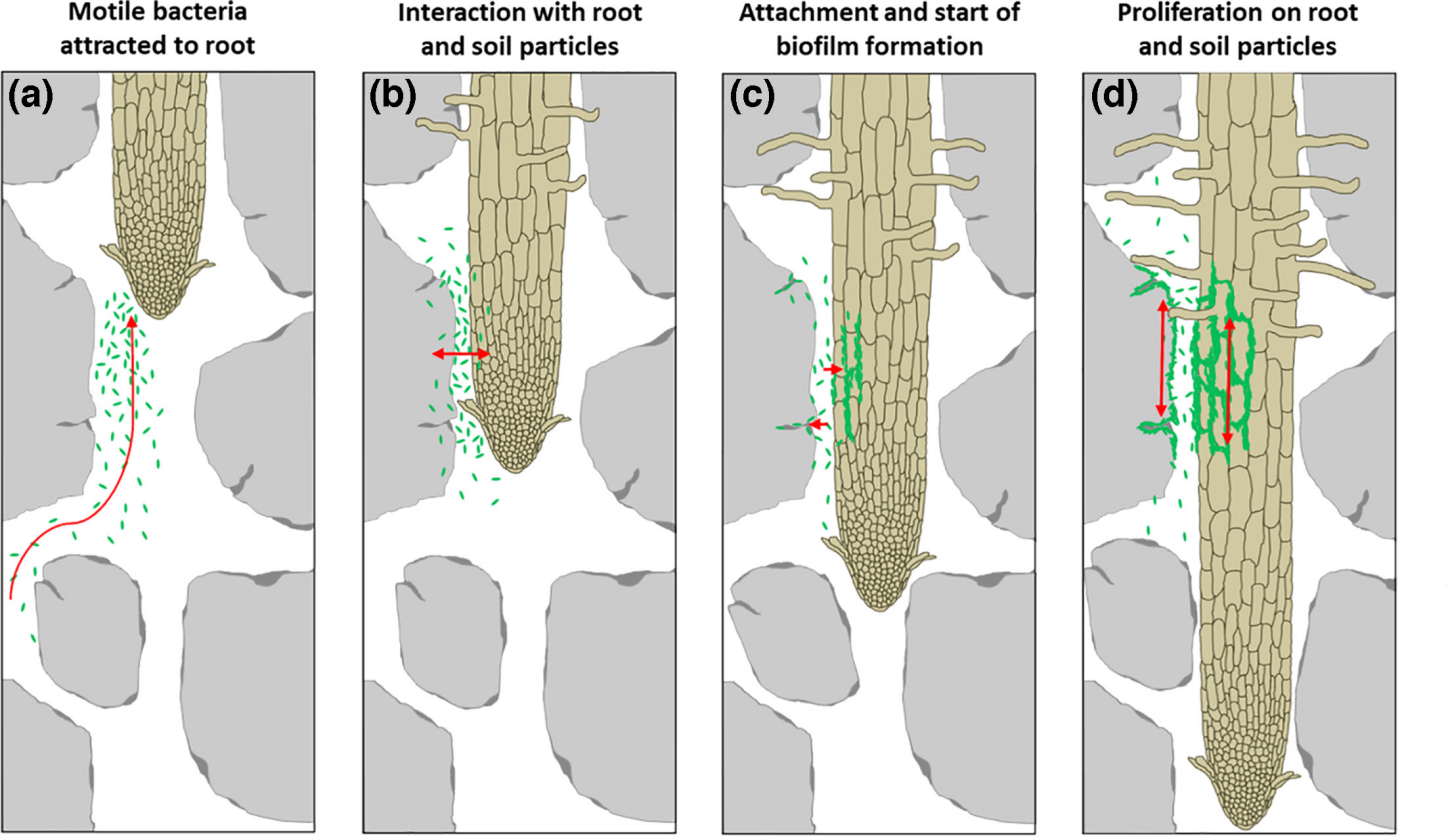
Diagrammatic representation of the root colonization over time. (**a**) MB navigate the pore space towards the root (nutrient source). (**b**) MB are particularly attracted to the confined spaces created by the soil particles and root surface, and they start to interact with the root surfaces at these hotpots. (**c**) Over time, the areas of high numbers of MB lead to attachment on roots (particularly in root cell junctions) and soil particle surfaces (particularly in crevices). (**d**) Attachment of groups of bacteria is followed by proliferation and formation of a mature biofilm along mature parts of the root but not on a growing root tip.

The first step required for colonization of the root, therefore, is the ability to navigate towards it through the highly complex soil matrix (Fig. 6a). It has been well established that microbes show enormous passive movement through dispersal [55] and that colonization is frequently driven by the large proliferation capabilities of microbial cells once within a suitable microhabitat [56] but also short-range co-ordinated collective movement over solid surfaces (e.g. swarming [57,58]), in liquid cultures (e.g. active fluids, [59]) or liquid-filled pore spaces [54]. Both active motility and chemotactic abilities have been shown to enhance the ability of *B. subtilis* to colonize roots efficiently [32,60], and previous work in a similar setting has shown, by means of calculation, that the even faster recruitment of cells through active motility and attraction [54] may also likely play a significant role in hotspots of biofilm formation along the root.

In a second step, the group movement of bacteria (shown as clouds in our image dataset) creates hotspots around the root (Fig. 6b). We observed such hotspots in areas of root–soil particle contact zones, likely high in root polysaccharides which fuel the microbes and may act as a cue to differentiate into matrix producing cells. Although at this point we still identify a high level of microbial activity near the root tip or in front of it, this activity may only be a precursor to groups of microbes entering in close proximity with sites favourable to colonization of the rhizoplane. At the sites of proximity between bacterial hotspots and the root surface, the mobile microbial cells can interact with the root surface, and an important third step (Fig. 6c) of the colonization likely consists cell-specific interactions between the bacterium and the root. Many rhizobacteria have known cell-specific interactions with plant roots [15]. In *B. subtilis*, proteins and polysaccharides are known to act as a cue to stimulate attachment [39] and biofilm formation [14,34].

The final step (Fig. 6d) following close proximity, contact and attachment is the permanent establishment of the bacterium on the root surface, most likely through the formation of biofilms. Biofilm formation is essential for *B. subtilis* to colonize plant roots. A large body of work exists describing in detail the machinery required, environmental cues and sequence of events for *B. subtilis* to form a stable biofilm on the root surface of a variety of plants. In short, cells are embedded in a matrix made up of exopolysaccharides (EPS) and proteins (tasA) which polymerize into amyloid-like fibres to make a protective microenvironment [61,62]. Consistent with previous work, we found that mature biofilm is typically found in the cell junctions and around root hairs of the more mature root zones. Even with our more pronounced hotspots of bacterial activity created by our complex matrix, we found that *B. subtilis* predominantly colonized these mature root zones, consistent with the previous findings for *B. subtilis* on a variety of plant roots [10,15, 35]. Mature root zones, especially the elongation zone, have been linked to particularly high exudation rates due to its immature endodermal barriers [63] and therefore are likely attractive for bacterial colonization.

## supplementary material

10.1099/mic.0.001477 Uncited Supplementary Material 1.

## Abbreviations

MB: mobile bacteria
PB: particle biofilm
RB: root biofilm
