# Supplementary material for: Mobility and growth in confined spaces are important mechanisms for the establishment of Bacillus subtilis in the rhizosphere
Source: Microbiology (Reading). 2024 Aug 6;170(8):001477. doi: 10.1099/mic.0.001477 (PMC11574552; doi:10.1099/mic.0.001477)
Supplement: Uncited Supplementary Material 1. [file mic-170-01477-s001.pdf]

Supplementary material

Mobility and growth in confined spaces are important mechanisms for the establishment of *Bacillus subtilis* in the rhizosphere

Ilonka C Engelhardt [1,2], Nicola Holden [3], Tim Daniell[4], Lionel Dupuy[2,5]

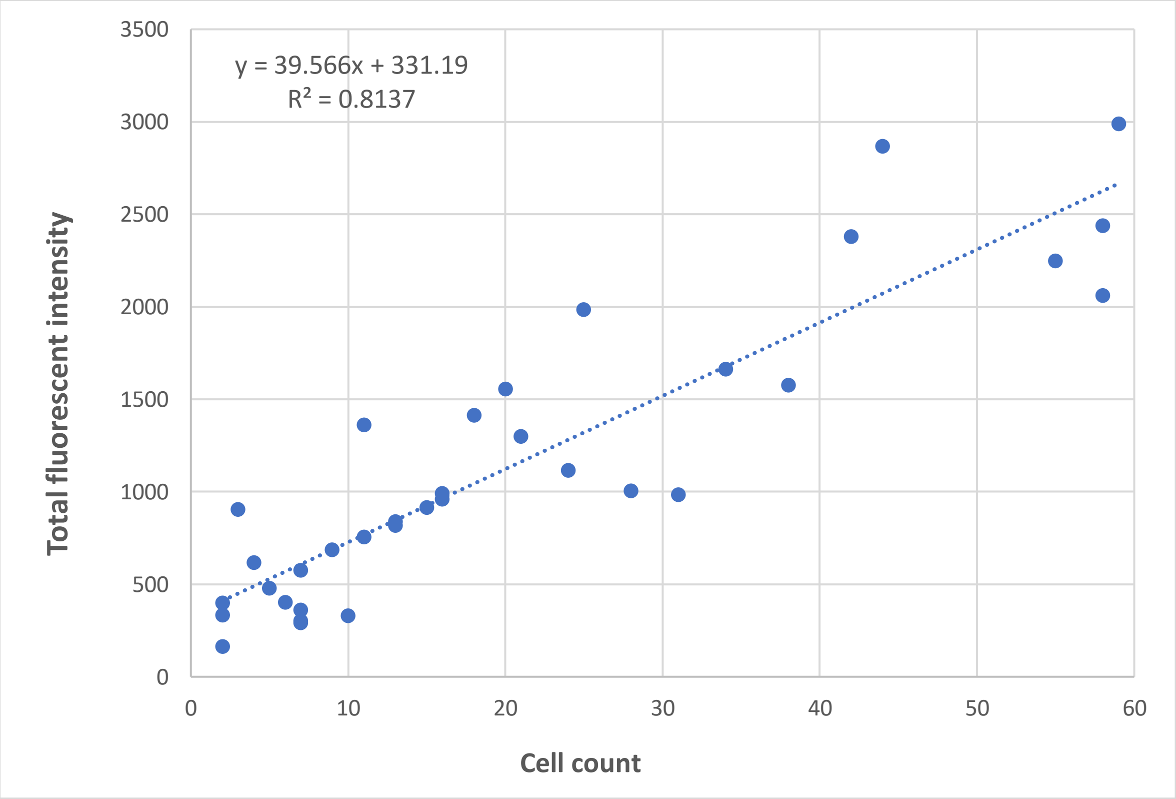

Fig S1. Correlation between total fluorescent intensity (mean fluorescence x area measured) and cell count. Each pixel over the predefined fluorescnece threshold was counted as 1 cell since the pixel size (6.25 x 6.25um) is roughly the same size as the average *B.subtilis* cell size (2-6 x 1um).

Table S1. Bacterial strains used in this study

Name	Genotype [a]	Reference / Construction [b]
NCIB 3610	Wild type prototroph	Bacillus genetic stock centre
NRS1473	NCIB 3610 *sacA*::Phy-spank-*gfp mut2* (kan)	(1)

[a] antibiotic resistance cassettes are indicated as follows: kan, kanamycin resistance

[b] A reference is provided if the strain or plasmid has previously been described and published.

1. Verhamme DT, Kiley TB, Stanley-Wall NR. DegU Co-Ordinates Multicellular Behaviour Exhibited by *Bacillus subtilis*. Mol Microbiol. 2007; 65 (2): 554–68.

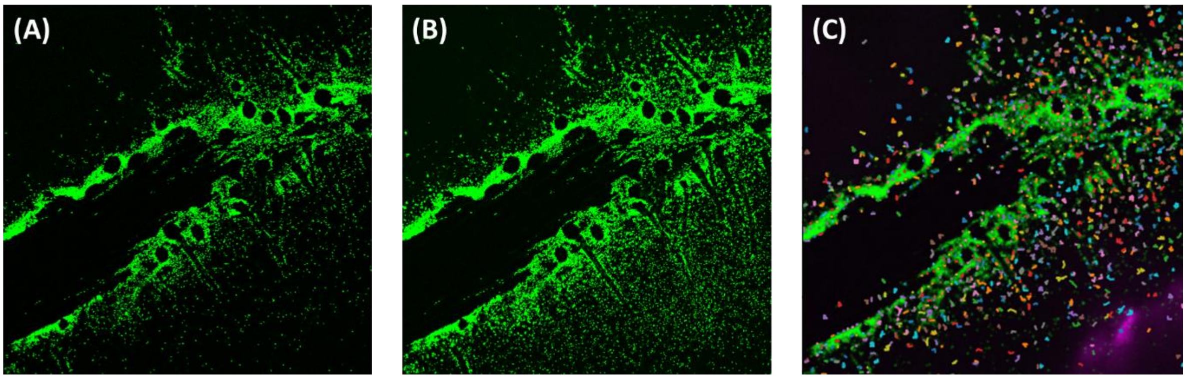

Figure S2. Motile bacteria and their appearance in . (A) A cross section obtained at a given timepoint obtained with a Nikon Fluor 20X W DIC microscope objective in a place of high cell density close to the root. Bacterial cell density is high on the root and root hairs and it is not possible to observe individual point representing a single cell. In the free space around the root, cell density is more sparce and single points likely represent the presence of a single cell. (B) When a max projection transformation of the data is applied to a root, here from a time lapse dataset but also when Z stacks were acquired in the rest of the study, moving bacteria in the pore space appear like a fuzzy cloud. (C) Using a tracking algorithm indicate some of the point observed in the cloud of mobile bacteria may correspond to the same bacteria.

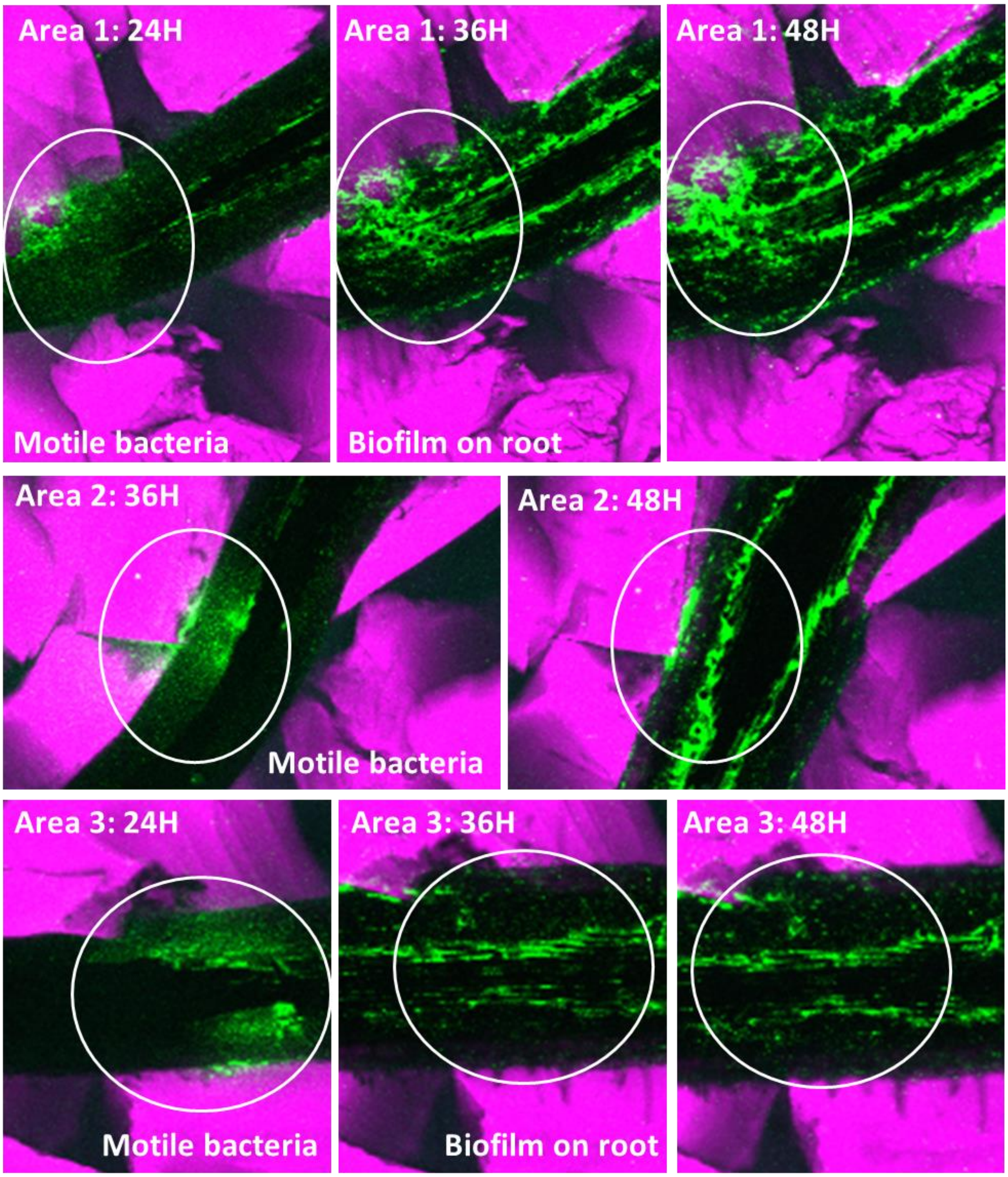

Figure S3. Selected areas on different roots over time show how motile bacteria in the vicinity of the root precede zones of biofilm formation on the root during the phases of colonisation.
